# Human Muscle Progenitor Cells Displayed Immunosuppressive Effect through Galectin-1 and Semaphorin-3A

**DOI:** 10.1155/2012/412610

**Published:** 2012-04-23

**Authors:** Séverine Lecourt, Yves Lepelletier, Valérie Vanneaux, Rafika Jarray, Thomas Domet, Françoise Raynaud, Réda Hadj-Slimane, Audrey Cras, Olivier Hermine, Jean-Pierre Marolleau, Jérôme Larghero

**Affiliations:** ^1^INSERM UMR940, Institut Universitaire d'Hématologie, 75475 Paris Cedex 10, France; ^2^Unité de Thérapie Cellulaire et CIC de Biothérapies, Hôpital Saint Louis, AP-HP, 75475 Paris Cedex 10, France; ^3^Université Paris Diderot, Sorbonne Paris Cité, 75475 Paris, France; ^4^CNRS UMR 8147, Hôpital Necker and Université Paris Descartes, 75743 Paris Cedex 15, France; ^5^Laboratoire de Chimie et Biochimie Pharmacologiques et Toxicologiques, CNRS UMR 8601, Université Paris Descartes, 75006 Paris, France; ^6^TRAGEX PHARMA, 75015 Paris, France; ^7^Service d'Hématologie Clinique, Hôpital d'Amiens, 80054 Amiens Cedex 1, France

## Abstract

In human skeletal muscle, myoblasts represent the main population of myogenic progenitors. We previously showed that, beside their myogenic differentiation capacities, myoblasts also differentiate towards osteogenic and chondrogenic lineages, some properties generally considered being hallmarks of mesenchymal stem cells (MSCs). MSCs are also characterized by their immunosuppressive potential, through cell-cell contacts and soluble factors, including prostaglandin E-2 (PGE-2), transforming growth factor-**β**1 (TGF-**β**1), interleukine-10, or indoleamine 2,3-dioxygenase. We and others also reported that Galectin-1 (Gal-1) and Semaphorin-3A (Sema-3A) were involved in MSCs-mediated immunosuppression. Here, we show that human myoblasts induce a significant and dose-dependant proliferation inhibition, independently of PGE-2 and TGF-**β**1. Our experiments revealed that myoblasts, in culture or *in situ* in human muscles, expressed and secreted Gal-1 and Sema-3A. Furthermore, myoblasts immunosuppressive functions were reverted by using blocking antibodies against Gal-1 or Sema-3A. Together, these results demonstrate an unsuspected immunosuppressive effect of myoblasts that may open new therapeutic perspectives.

## 1. Introduction

Human satellite cells, positioned under the basal lamina, have been identified to be the main myogenic progenitor undergoing activation, expansion into myoblasts, and self-renewal [1, 2]. The surface cell antigen CD56 has been considered as a specific marker for cells derived from muscle satellite cells [3]. We recently showed that CD56+ myoblasts are able to differentiate into myotubes but also into osteoblasts and chondroblasts [4]. The ability to differentiate towards osteogenic and chondrogenic lineages is considered to be a functional characteristic of mesenchymal stem cells (MSCs) [5]. Beside these differentiation potentials, MSCs have been shown to exert an immunosuppressive role on T and B lymphocytes, natural killer and dendritic cells [6–11]. While the mechanisms that govern this immunosuppressive activity are still a matter of debate, several studies have reported the role of cell-cell contact and soluble factors [12]. Recently, we and others showed that MSCs exert suppressive effect on T cell through two soluble factors, Galectin-1 (Gal-1) and Semaphorin-3A (Sema-3A) [13–15]. Gal-1 and Sema-3A are two ligands able to bind to Neuropilin-1 (NP-1), a neuronal receptor constitutively expressed on T-cell surface and involved in the regulation of T-cell proliferation [16]. In muscle environment, Gal-1 promotes myoblast fusion and axonal growth after muscle injury. Sema-3A is expressed by satellite cells in injured muscle in response to hepatocyte growth factor secretion and is involved in the control of myofiber innervation [17, 18]. In this context, we aimed to investigate the potential immunosuppressive function of myoblasts and to determine the mechanisms by which they exerted this function. We showed that human myoblasts, similarly to MSCs, have immunosuppressive properties on PBMCs. Both Gal-1 and Sema-3A were expressed and secreted by myoblasts. These secreted proteins have largely been identified as immunosuppressive factors controlling T-cell proliferation. Our data revealed that inhibition of PBMCs proliferation was driven by Gal-1 and Sema-3A, thus demonstrating that these two soluble factors mediate the myoblasts immunosuppressive effect.

## 2. Material and Methods

### 2.1. Cell Culture

Muscle biopsies were obtained via the Tissue Bank for Research of the French Association against Myopathies, upon informed consent. Biopsies were 0.3–4 g res nullus specimen from orthopaedic surgery. The three donors were adults and had no clinical signs of muscular disease. Muscle biopsies were enzymatically dissociated and cells cultured in proliferation medium promoting the expansion of CD56+ myogenic cells, as previously described [19]. MSCs were isolated from washed filters used during bone marrow (BM) graft processing for allogenic BM transplantation (*n* = 3). MSCs were obtained, phenotyped, and cultured as previously described [20]. Myoblasts and MSCs were used at passage 2 or 3.

### 2.2. Cell Characterization

Myoblasts were stained with the following FITC or PE-conjugated antibodies: anti-Gal-1, -Sema-3A, -CD56, -desmin, -CD44, -CD45, -CD80, -CD86, -CD105, -HLA Class I, -HLA Class II, or with appropriate controls and analyzed using a FACScalibur (Becton Dickinson, Le Pont de Claix, France). Immunoprecipitation were performed using Sema-3A- and Gal-1-specific antibodies detected by HRP-conjugated antibody and revealed with ECL kit (Thermo Fisher Scientific, Brebières, France).

Myoblasts differentiation into myotubes was evaluated by immunofluorescence staining with myosin heavy chain antibody (Ozyme, Saint Quentin en Yvelines, France).

### 2.3. Mixed Leucocyte Reaction

Peripheral blood mononuclear cells (PBMCs) were isolated from res nullus of apheresis product after Ficoll gradient separation. PBMCs from a normal donor were mixed with irradiated CD56+ cells (25 Gy) and PBMCs from another healthy individual as previously described at concentrations ranging from 0.1 to 20% of PBMCs/wells (i.e., 100 to 20,000 CD56+ cells) [20]. PBMCs from a total of 8 healthy donors were used in these experiments. After 5 days of incubation, 1 *μ*C of  ^3^H-thymidine (Amersham, Les Ulis, France) was added overnight and thymidine incorporation was measured using a *β*-scintillation counter (Beckman, Gagny, France) and expressed as counts per minute (cpm). The percentage of inhibition was calculated as follows: 100-(cpm MLR + MSCs/cpm MLR) ∗ 100.

For blocking experiment, CD56+ cells were seeded at the concentration of 20% of responder PBMCs and were incubated with neutralizing Gal-1 and Sema-3A antibodies (10 to 40 *μ*g/mL, generous gift from Dr. A. Shirvan and Dr. A. Barzilai).

### 2.4. Soluble Factors Production

TGF-*β*1 levels in myoblasts culture supernatants were assessed by enzyme-linked immunosorbent assay (ELISA) using commercially available kits (R&D Systems, Lille, France) according to manufacturer's instructions. Quantitative analysis of PGE2 was performed using a competitive binding technique according to the manufacturer's protocol (R&D systems).

### 2.5. RT-PCR

CD56+ cell RNA was extracted with NucleoSpinRNA II (Macherey-Nagel, Hoerd, France). One *μ*g of each RNA sample was reverse-transcripted into cDNA using iScript cDNA Synthesis Kit (Bio-Rad, USA). Specific primers used were Gal-1, 5′-GGT-CGC-CAG-CAA-CCT-GAA-TC-3′, and 5′-ATG-TAG-TTG-ATG-GCC-TCC-AG-3′; Sema-3A, 5′-AGA-CGC-ACA-AGA-CGA-CAA-GA-3′ and 5′-GCC-TTG-ATC-TGT-CCT-GAT-GAT-3′; RPLO (ribosomal protein L15), 5′-CAT-TGC-CCC-ATG-TGA-AGT-C-3′ and 5′-GCT-CCC-ACT-TTG-TCT-CCA-GT-3′. PCR products were analyzed by using Gel Doc 2000 System (Bio-Rad, USA).

### 2.6. Immunofluorescence

Muscle samples were fixed in 4% paraformaldehyde, quenched with 0.1 M glycine, incubated in permeabilizing buffer containing 0.1% triton and anti-Gal-1 or anti-Sema-3A antibodies and revealed using alexa488- or alexa547-secondary antibodies (Invitrogen, Saint-Aubin, France). Nucleus was stained using Dapi (Invitrogen). Mounted slides were scanned with a Nikon Epifluo TE-2000E microscope (Nikon Instruments Europe) and were subsequently analyzed with Image J software.

### 2.7. Statistical Analysis

The results were analyzed by independent sample two-tailed and unpaired Student's *t*-test and were presented as means ± standard error deviation.

## 3. Results and Discussion

**Figure 1:**
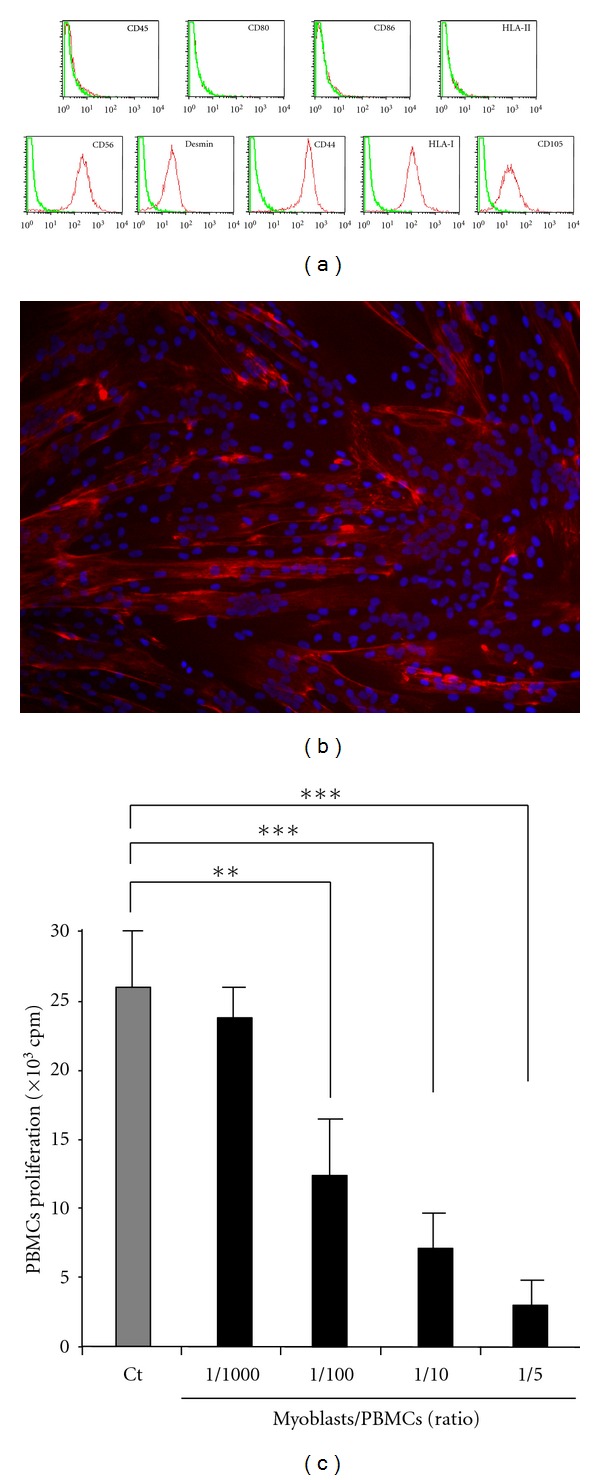
Human myoblasts characterization and PBMCs proliferation inhibition. (a) Immunophenotyping by flow cytometry analysis: cells were labeled with specific antibodies (red line) or isotype control (green line). (b) Myogenic differentiation ability of CD56+ cells. Myotubes expressed myosin heavy chain (red), nuclei were labeled with DAPI (blue). (c) PBMCs were cultured in the presence of increasing number of myoblasts at ratio ranging from 1/1000 to 1/5. Data are means ± SEM of triplicates from one representative of at least 10 independent experiments. *P* values from Student's *t*-test are indicated (**P < 0.01, ***P < 0.001).

Phenotypically, myoblasts were shown to express CD56, Desmin, CD44, CD105, and HLA class I, while they were negative for the expression of CD45, HLA class II, CD80, and CD86 (Figure 1(a)). On a functional point of view, as expected, myoblasts were shown to differentiate into myotubes (Figure 1(b)). In the present study, we demonstrated for the first time that human myoblasts have immunosuppressive functions on PBMCs *in vitro*. Similarly to MSCs isolated from BM, synovium or adipose tissues, myoblasts were able to inhibit PBMCs proliferation in a mixed-lymphocyte reaction [7, 21, 22]. This inhibition was dose dependent, with PBMCs proliferation inhibition being more efficient at higher myoblasts:PBMCs ratio (Figure 1(c)).

**Figure 2:**
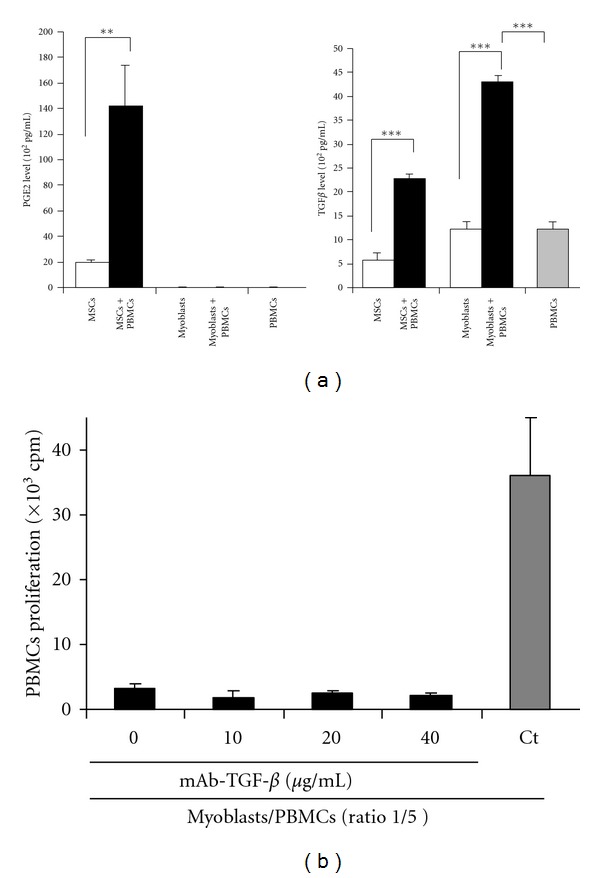
Human myoblasts inhibit PBMCs proliferation independently of PGE-2 and TGF*β*-1. (a) PGE-2 (left panel) and TGF-*β* (right panel) levels were assessed by ELISA on culture supernatants of MSCs and myoblasts, in the presence (black bars) or absence (white bars) of PBMCs. (b) TGF-*β*1 blocking antibodies (10 to 40 *μ*g/mL) were used to neutralize the immunosuppressive activity of myoblasts on PBMCs. PBMCs and myoblasts were cultured at a ratio 1/5.

While the mechanisms by which MSCs exerted their immunosuppressive effects have not been completely elucidated to date, several soluble factors have been described as key regulators of this process. TGF-*β*1 and PGE-2 have been precociously reported to participate in the MSCs-mediated regulation of immune responses [7, 23]. Compared to MSCs, myoblasts immunosuppressive properties were shown to be independent of PGE-2 and TGF-*β*1. Myoblasts, alone or in the presence of PBMCs, do not secrete PGE-2 (Figure 2(a), left panel). Contrarily, myoblasts were found to secrete high basal levels of TGF-*β*1. Furthermore, TGF-*β*1 secretion was significantly increased when MSCs or myoblasts were cultured in the presence of PBMCs, (Figure 2(a), right panel). However, no significant reversion of myoblasts immunosuppressive effect was observed on PBMCs proliferation in the presence of TGF-*β*1 blocking antibodies (Figure 2(b)).

**Figure 3:**
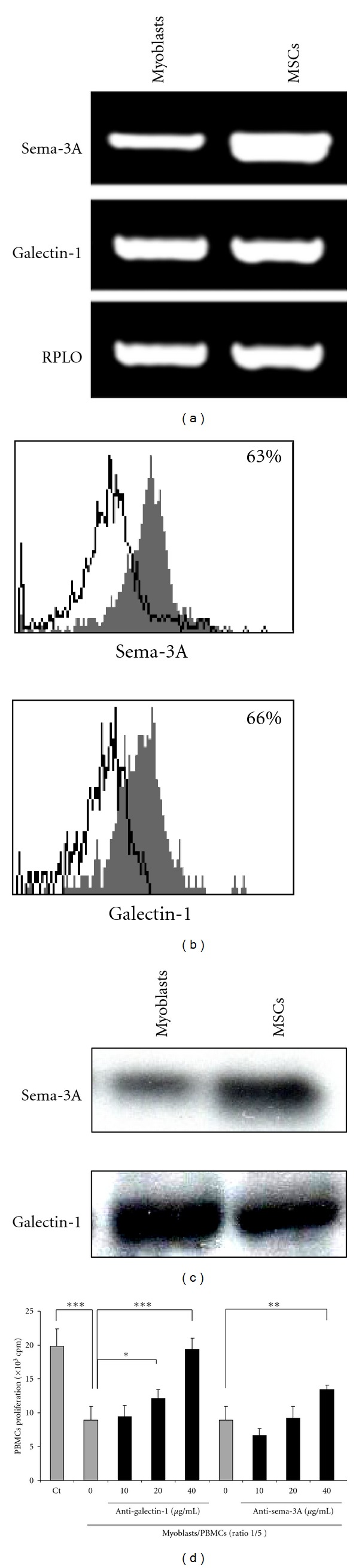
Human myoblasts immunosuppression is mediated by Gal-1 and Sema-3A. (a, b, c) RT-PCR, flow cytometric, and immunoprecipitation analysis of Gal-1 and Sema-3A expression and secretion by myoblasts. MSCs were used as control. Data are means ± SEM of triplicates from one representative of at least 10 independent experiments. *P* values from Student's *t*-test are indicated (**P < 0.01, ***P < 0.001). (d) Neutralization of Gal-1 and Sema-3A restores PBMCs proliferation. PBMCs from eight independent donors were cultured alone (A+B) or with myoblasts (ratio 1/5) in the presence of increasing concentration of anti-Gal-1 or anti-Sema-3A antibodies (0 to 40 *μ*g/mL). Data are means ± SD of triplicates from one representative of at least 10 independent experiments. *P* values from Student's *t*-test are indicated (*P <0.05, **P < 0.01, and ***P < 0.001).

Gal-1 and Sema-3A are two soluble factors with immunosuppressive function acting through NP-1 expressed on T cells [24, 25]. Our recent work showed that both Gal-1 and Sema-3A were highly expressed by MSCs, conferring to these cells a suppressive activity on PBMCs proliferation [13]. We demonstrated that myoblasts expressed both these molecules at the mRNA (Figure 3(a)) and protein level (Figure 3(b)). Gal-1 and Sema-3A were also secreted, as shown by immunoprecipitation of the myoblasts culture supernatant (Figure 3(c)). 

**Figure 4:**
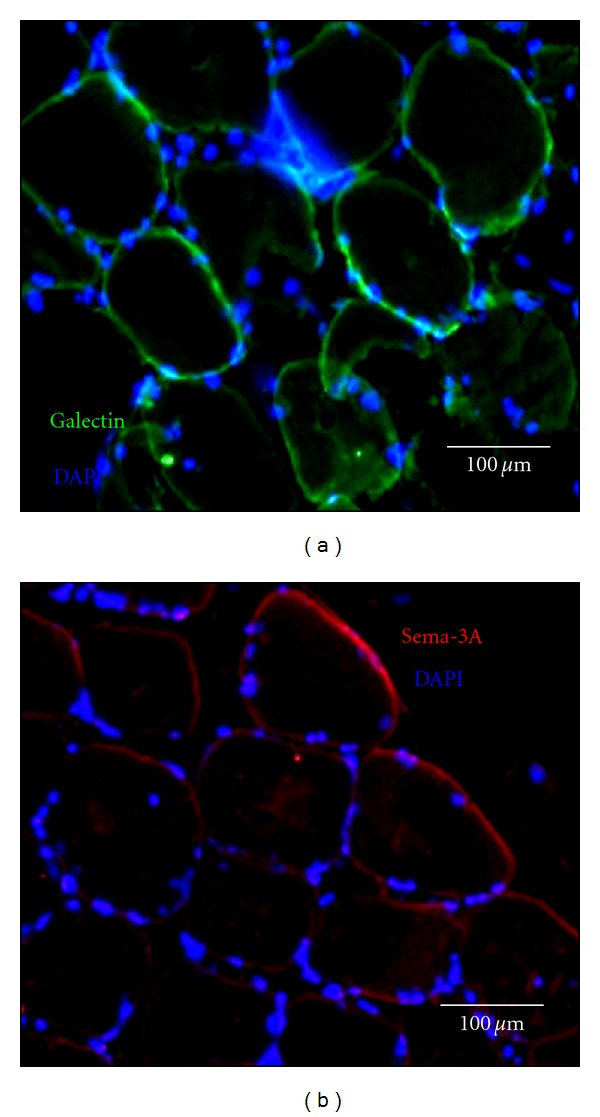
*In situ* Gal-1 and Sema-3A expression. (a) Microscopy analysis of Gal-1 (green) expressions *in situ* in normal human muscle. Bar represents 100 *μ*m. (b) Microscopy analysis of Sema-3A (red) expressions *in situ* in normal human muscle. Bar represents 100 *μ*m. Nuclei were stained with Dapi and appeared in blue.

Interestingly, myoblasts secreted higher amount of Gal-1 than of Sema-3A, contrarily to that was observed for MSCs, used as a control. We next assessed whether Gal-1 and Sema-3A were directly involved in myoblasts-mediated PBMCs immunosuppression, since both ligands are capable to specifically inhibit PBMCs proliferation. To this aim, myoblasts and PBMCs were cocultured in the presence of neutralizing Gal-1 and Sema-3A antibodies. The blockade of Gal-1 and Sema-3A, secreted by myoblasts, restored the proliferation of PBMCs in a dose-dependent manner (Figure 3(d)). Consistently with the amounts of both molecules secreted by myoblasts, the restoration of PBMCs proliferation was higher with anti-Gal-1 antibodies than with anti-Sema-3A antibodies (98% and 68% at 40 *μ*g/mL, resp.). Taken together, these results demonstrated the implication of Gal-1 and, at a lesser extent of Sema-3A, in the immunosuppressive properties of myoblasts. In order to evaluate the relevance of these findings *in situ*, Gal-1 and Sema-3A expressions were assessed on human muscle biopsies. Similarly to that was observed on cultured myoblasts, both molecules were expressed in healthy human muscles (Figures 4(a), 4(b)). Gal-1 expression was greater than that of Sema-3A, consistently with the *in vitro* observations.

Emerging findings have pointed out the active interactions between muscle cells and the immune system. Muscle cells secrete cytokines involved in immune and inflammatory reactions, respond to inflammatory molecules, and may act as antigen-presenting cell in particular conditions [26]. Moreover, it has been shown that, in inflammatory myopathies, muscle cells expressed costimulatory molecules, cell adhesion molecules, and cytokine receptors [27–29]. Our study is limited due to the lack of experiment on myoblasts from pathological conditions. Acknowledging these limitations, our data indicate that the Sema-3A- and Gal-1-mediated immunomodulation may contribute to protect muscle from inflammatory and/or immune injuries.
